# Genetic Population Structure of Wild Boars (*Sus scrofa*) in Fukushima Prefecture

**DOI:** 10.3390/ani12040491

**Published:** 2022-02-16

**Authors:** Rie Saito, Natsuko Ito Kondo, Yui Nemoto, Reiko Kumada, Nobuyoshi Nakajima, Masanori Tamaoki

**Affiliations:** 1 Fukushima Prefectural Centre for Environmental Creation, 10-2 Fukasaku, Miharu-machi 963-7700, Japan; kumada_reiko_02@pref.fukushima.lg.jp; 2 Fukushima Regional Collaborative Research Center, National Institute for Environmental Studies, 10-2 Fukasaku, Miharu-machi 963-7700, Japan; mtamaoki@nies.go.jp; 3 Biodiversity Division, National Institute for Environmental Studies, 16-2 Onogawa, Yamagata 305-8506, Japan; kondo.natsuko@nies.go.jp (N.I.K.); naka-320@nies.go.jp (N.N.); 4 Okutama Practice Forest, Tokyo University of Agriculture, Hikawa 2137, Okutama 198-0212, Japan; yui.wildlife@gmail.com

**Keywords:** wild boars, MIG-seq analysis, genetic population structure, single-nucleotide polymorphisms, Fukushima Prefecture

## Abstract

**Simple Summary:**

After the Fukushima Daiichi nuclear power plant accident, a highly contaminated area with radionuclide appeared and was designated a difficult-to-return zone (DRZ). After that, the increase in number of wild boars (*Sus scrofa*) has been pronounced in DRZ, and the spread of highly contaminated wild boars into other areas is cause for concern. Understanding the population structure based on the genetic diversity of wild boars in Fukushima provides important information for the management of the animals. In this study, we carried out MIG-seq analysis to clarify the dispersal and gene flow of the local wild boar population and uncover the genetic population boundary in Fukushima. We obtained 328 single-nucleotide polymorphisms from 179 wild boars. Based on STRUCTURE analysis, we found significant genetic differences between groups of wild boars inhabiting in the east and west, divided by the Abukuma River. Since the urbanized area is concentrated along the Abukuma River in Fukushima, both the Abukuma River and the urbanized area are likely to interfere in the migration and dispersal of wild boars. Furthermore, our results indicate that the population in the western area was established by the migration from other neighboring prefectures rather than by that from the eastern group of Fukushima Prefecture.

**Abstract:**

We aimed to reveal the dispersal and gene flow of the local wild boar (*Sus scrofa*) population and find their genetic boundary in Fukushima Prefecture. After the nuclear incident in 2011, the land was considered a difficult-to-return zone, and the increase in the number of wild boars was pronounced. To provide an effective management strategy for the wild boar population, we used multiplexed inter-simple sequence repeat genotyping by sequencing (MIG-seq) and clarified the genetic structure of wild boars. We obtained 328 single-nucleotide polymorphisms from 179 samples. STRUCTURE analysis showed that the most likely number of population cluster was *K* = 2. Molecular analysis of variance showed significant genetic differences between groups of wild boars inhabiting in the east and west across the Abukuma River. The migration rate from the eastern population to the western population is higher than in the reverse case based on BayesAss analysis. Our study indicates that both the Abukuma River and anthropogenic urbanization along the river may affect the migration of wild boars and the population in western was established mainly by the migration from other neighboring prefectures.

## 1. Introduction

**Figure 1:**
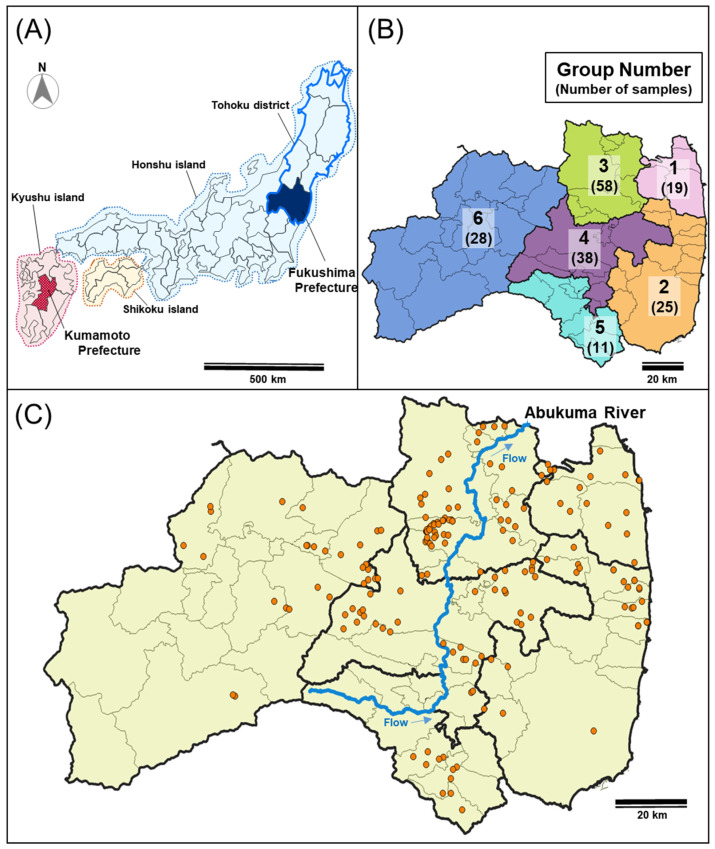
Sampling locations of wild boars. (**A**) Location of Fukushima Prefecture and Kumamoto Prefecture. (**B**) Six regions of Fukushima Prefecture (1, North of Soso; 2, South of Soso and Iwaki; 3, Ken-Poku; 4, Ken-Chu; 5, Ken-Nan; 6, Aizu). Numbers in parentheses represent the number of wild boars analyzed in each district region. (**C**) The sampling location of wild boars in Fukushima Prefecture. Sampling points of wild boars are shown in orange dots. These figures were created using QGIS 3.1.6 (https://www.qgis.org/en/site/, accessed on 15 February 2022). The map of Fukushima Prefecture and Abukuma River were obtained by Ministry of Land, Infrastructure, Transport and Tourism (MLIT) of Japan (http://nlftp.mlit.go.jp/ksj/, accessed on 15 February 2022).

Recently, some mammals, such as deer and wild boar, have expanded their population size and their habitats expand closer to human dwellings, resulting in damage to agriculture, forestry, and ecosystems in Japan [1,2]. Wild boars are naturally distributed in Asia, Europe, and northern Africa [3], and two subspecies are distributed in Japan as follows: The Japanese wild boar (*Sus scrofa leucomystax*) and the Ryukyu wild boar (*Sus scrofa riukiuanus*). The Japanese wild boar (hereafter referred to as “wild boar”) is widely distributed in mainland Japan, including Honshu, Shikoku, and Kyushu islands, except for the north Tohoku district [4] (Figure 1). Wild boars have been targeted for hunting and are preferred as a food source locally, but they also cause serious damage to crops. Therefore, wild boars have suffered from increased hunting pressure to prevent agricultural damage since the Meiji era (i.e., 1968–1912), and their habitats have decreased due to increased urbanization. Moreover, swine fever, which has a severe impact on the pig industry as well as wild boar populations, became epidemic in Japan [5]. These resulted in the reduction of wild boar populations in Japan until the 1980s [5,6].

In recent years, the number of wild boars has rapidly increased as a result of increased abandoned land accompanied by the decline in human activity around the rural area (i.e., Satoyama area) and the reduction of hunting pressure due to the aging population [7,8]. In addition, wild boars have expanded their habitats via re-invasion in certain regions, such as north Tohoku, where they could not inhabit over the winter previously due to heavy snowfall [9,10]. In Japan, wild boars caused 4733 million yen worth of damage to crops in the 2018 fiscal year, which is the second largest animal-caused damage—just behind deer. The amount of damage caused by wild boars was four times that of birds or other animals, such as monkeys, in the 2018 fiscal year [11]. The increase in the population of wild boars has also become a major problem in Fukushima Prefecture. Distribution surveys conducted before 2002 showed that wild boars only inhabited the eastern Fukushima Prefecture [5]. However, wild boars were found in the western Fukushima Prefecture, upon surveillance in 2004, suggesting that the wild boars habitat in Fukushima Prefecture have expanded rapidly within recent years [5]. Specific to wild boars in Fukushima Prefecture, the effects of the accident at the Tokyo Electric Power Company Fukushima Daiichi Nuclear Power Plant (FDNPP, Figure S1A) in 2011 were particularly notable. After the accident, radioactive cesium above the allowed limits for radionuclides in food was detected in wild boars in Fukushima Prefecture, resulting in the restriction of the ingestion or commercial distribution of wild boar meat caught in Fukushima Prefecture. This decreased the motivation for hunting wild boars in Fukushima Prefecture. Moreover, a part of Fukushima Prefecture close to the FDNPP was designated as an evacuation zone (i.e., difficult-to-return zone (DRZ), Figure S1A) due to a high level of radiation, and wild boar numbers are higher within this evacuation zone compared to that outside the zone [5,12]. Furthermore, the activity range of wild boars expanded in Fukushima evacuation zone, and it is a concern that wild boars containing high concentrations of radionuclides may be dispersed to other areas. In general, population management for wild boars is mainly conducted under the control of the prefecture or each municipal unit, but wild boars move and disperse beyond the boundaries of municipalities. To manage the population of wild boars, it is important to collect information on the habitat and ecological characteristics beyond the boundaries of municipalities [6].

Understanding the population structure based on the genetic diversity and genetic boundary of species is important for wildlife management [13]. The genetic structure of populations mainly reflects the migration and dispersal in nature [14,15]. Studies on the genetic population structure based on DNA analysis have been previously conducted on wild boars to determine the background of their geographical distribution [13,16,17,18,19,20,21,22]. Based on changes in base sequences of mitochondrial DNA (mtDNA) control regions (D-loops) of wild boars inhabiting a wide area in Japan extending from Honshu island to Kyushu island inferred that geographical barriers, such as the Japanese Alps, restrict gene flow among local populations [22]. In addition, microsatellite analysis has also been used to determine the local-scale genetic population structure of wild boars [18,21]. For example, a study of wild boars based on both mtDNA sequence analysis and microsatellite analyses in the Tochigi Prefecture showed that microsatellite analysis represents a more recent population structure than mtDNA sequence analysis [18].

The use of microsatellite analysis has increased linearly since the early 1990s, whereas the use of genome-wide single-nucleotide polymorphisms (SNPs) has increased exponentially since the late 1990s [23]. Genetic population analysis based on the detection of genome-wide SNPs has many advantages in comparison to microsatellite analysis for understanding population structure as follows: a lower number of samples is required for an accurate estimation of allelic frequencies because of the large number of alleles per locus in microsatellite; SNPs are suitable for estimation of long-term population history due to the lower rate of recurrent or backward mutations; variability of highly polymorphic microsatellite markers may not accurately reflect the underlying genomic diversity [23]. High-throughput sequencing technologies allow the identification of large numbers of SNPs at reduced cost in non-model species. Multiplexed inter-simple sequence repeat genotyping by sequencing (MIG-seq) on non-model animals, plants, and fungi was performed, which identified the population structure within each species, demonstrating that this method can be applicable for population analysis in a wide group of taxa [24]. In this study, we carried out MIG-seq analysis to clarify the genetic population structure of wild boars in Fukushima Prefecture, in which the unusual DRZ has raised concerns and the increase in the numbers of wild boars has been observed after the FDNPP accident. This study aimed to reveal the dispersal and gene flow of the local wild boar population and find the genetic boundary for wild boars in Fukushima Prefecture, including the DRZ (Figure 1). This information will be useful for the management of wild boars in the area after the FDNPP accident.

## 2. Materials and Methods

### 2.1. Sample Collection

Since the FDNPP accident in 2011, Fukushima Prefectural government has been collecting meat fragments of wild boars from hunters to monitor the concentration of radioactive cesium in the muscles of wild boars. These boars were caught by hunters as part of efforts to control harmful wildlife implemented under the Wildlife Protection and Hunting Management Law (Law No. 32, 1918). As in the case of wild boars in DRZ, we used muscle samples of wild boars captured by the Ministry of the Environment under “The habitat survey and capture of the wild animals project in and around the former restricted areas (areas within 20-km radius from Fukushima Daiichi NPP)”, and “The habitat survey and capture of the wild animals project in and around the former restricted areas (areas within 20-km radius from Fukushima Daiichi NPP)”. Therefore, no wild boars were killed specifically for this research and no live animals were used. One hundred and seventy-nine wild boars captured in Fukushima Prefecture from October 2013 to June 2018 were used (Table S1). Information on the capture sites of wild boars was obtained from maps or addresses submitted by hunters. As for a comparative sample to wild boars in Fukushima Prefecture, nine samples of wild boars caught in the Kumamoto Prefecture in the Kyushu district were provided by Munemasa Kosan Co., Ltd. (http://shop.amakusa-web.jp/cinghiale, accessed on 15 February 2022) (Figure 1A). Kumamoto Prefecture is located approximately 1040 km away from Fukushima Prefecture and the Kyushu district is only connected by a bridge to Honshu island including the Tohoku district. The samples were analyzed in two ways based on the capture site of the wild boars as follows: One group was divided into seven regions (six regions in Fukushima Prefecture and one region in the Kumamoto Prefecture) (Table S1, Figure 1B); the other was divided into two groups (captured in the eastern and western Abukuma River, Table S1).

### 2.2. MIG-Seq Analysis

DNA was extracted from frozen, freeze-dried, or ethanol-fixed wild boar meat pieces. Genomic DNA was extracted using DNeasy EZ1 Kit (QIAGEN, Hilden, Germany) and BIO ROBOT EZ1 (QIAGEN). MIG-seq analysis was performed as previously described by Suyama and Matsuki [24]. For the preparation of the MIG-seq library, multiplex PCR was performed with eight forward and reverse primer sets using the MIG-seq primer set-1 for the first PCR [24]. The first PCR was conducted using Multiplex PCR Assay Kit Ver.2 (Takara Bio, Kusatsu, Japan), and contained 3.5 μL of 2× Multiplex PCR Buffer, 0.035 μL of Multiplex PCR Enzyme Mix, 1.0 μL of template DNA, and 0.2 μM of each primer. The volume of the reaction solution was adjusted to 7.0 μL with Nuclease Free Water. PCR conditions were as follows: Initial denaturation at 94 °C for 1 min, followed by 25 cycles of heat denaturation at 94 °C for 30 s, primer annealing at 48 °C for 1 min, extension reaction at 72 °C for 1 min, and final extension reaction at 72 °C for 10 min. The first PCR was performed three times per sample to detect more mutations. Equal amounts of the first PCR products were mixed from the three replicates, and the PCR products were purified using AMPure XP (Beckman Coulter Life Sciences, San Jose, CA, USA). The purified PCR product was used as the template for the second PCR to add the adaptor for Illumina MiSeq (Illumina, San Diego, CA, USA) and the Index for sample identification. We used a single index that was different for each sample as published by Matsuki and Suyama [24]. The second PCR was conducted using PrimeSTAR GXL buffer (Takara Bio, Kusatsu, Japan), and the PCR reaction solution consisted of 3.0 μL of 5× PrimeSTAR GXL buffer, 1.2 μL of 2.5 mM dNTP mixture, 1.0 μL of template DNA, 0.375 U of PrimeSTAR GXL Polymerase, 3.0 μL of purified first PCR product, and primers at a final concentration of 0.2 μM each, and the reaction solution volume was adjusted to 15.0 μL with Nuclease Free Water. PCR conditions were as follows: Heat denaturation at 98 °C for 10 s, primer annealing at 54 °C for 15 s, and elongation at 68 °C for 1 min for 12 cycles. The second PCR products were purified using the QIAquick PCR Purification Kit (QIAGEN) according to the manufacturer’s protocol. Size selection was performed using SPRI Size Select (Beckman Coulter Life Sciences, San Jose, CA, USA) to obtain 300–800 bp libraries. However, since fragments larger than 10,000 bp remained after the size selection with SPRI Size Select, the PCR products were electrophoresed on an agarose gel. Then, 300–800 bp fragments were cut out from the agarose gel and purified using the QIAquick Gel Extraction Kit (QIAGEN). Size distribution and concentration of the library were analyzed by Agilent 2200 TapeStation using the Genomic DNA ScreenTape System. Sequencing was performed by MiSeq (Illumina) using MiSeq Regent v3 150 cycle. FASTX-Toolkit ver.0.0.14 (http://hannonlab.cshl.edu/fastx_toolkit/, accessed on 15 February 2022) was used to remove the primer sequences (Minimum quality score to keep (q) = 33, Minimum percent of bases that must have “q” quality (p) = 40) from the raw reads. TagDust v2.31 [25] was then used to remove short reads containing primer sites in the sequence, and then Read1 and trimmed Read2 data were combined. At this point, the average number of reads for each sample was 482,605.6 (minimum 248,840 reads). SNPs were detected using Stacks v. 1.35 [26]. To create “stacks”, ustacks was used and set to a minimum depth of coverage (m) as 5 and maximum distance allowed between stacks (M) as 2. We used ctacks to catalog the data under mismatches between sample loci (*n*) as 4, and then used sstacks to determine the SNP loci for each individual. We used stacks to select SNPs with a minimum percentage of samples in a population (r) as 0.5 and the minimum number of populations in a locus (p) as 2; we set up separate populations for individuals captured in Fukushima and Kumamoto prefectures. For the parameters not specifically mentioned above, all default values were used. For the candidate SNP loci obtained, loci that appeared only once in all individuals were excluded from further analysis. CLC Genomic Workbench (CLC bio, Aarhus, Denmark) was used to perform pairwise similarity calculations after the alignment of sequences containing SNPs. SNPs showing more than 50% sequence similarity were considered duplicates and removed, and the remaining SNP loci were extracted. The registration number of the obtained sequences are in Table S1.

### 2.3. Data Analysis

Genetic population structure estimation based on the obtained SNPs was performed using STRUCTURE ver. 2.3.4 [27]. We set the first burn-in period 100,000 times and then performed 150,000 calculations using the Markov chain Monte Carlo (MCMC) method. The number of clusters (*K*) was set from 1 to 6, and ten calculations were performed for each *K*. After STRUCTURE analysis, we calculated Δ*K* [28] using Structure Harvester ver. 0.6.94 [29], and the value of *K* with the highest Δ*K* was set as the optimal number of clusters. STRUCTURE analysis was performed independently for each data set of wild boars from the six populations of Fukushima and Kumamoto prefectures or only wild boars from six populations of Fukushima Prefecture. Hierarchical cluster analysis was performed for six groups in Fukushima Prefecture via Ward’s method using “stat” program that is implemented in R package by default (R 4.1.0; https://cran.r-project.org/bin/windows/base/, accessed on 15 February 2022), then tree diagrams were created. The fixed index of genetic differentiation (*Fst*) among the six groups in Fukushima Prefecture was calculated using AMOVA approach in GenAlEx 6.503 [30]. AMOVA was conducted on the SNPs for eastern and western Abukuma River groups using GenAlEx 6.503 [30] to examine the partition of genetic variation among the groups. Statistical significance of calculation of pairwise *Fst* values and AMOVA was assessed based on 9999 permutations and applied the “interpolate missing” option. *p*-Values of pairwise *Fst* values were corrected for multiple comparisons using the Bonferroni method [31]. We estimate migration rates between eastern and western Abukuma River groups using the MCMC method in BayesAss 3.0.4 [32]. Preliminary estimation were conducted to adjust the acceptance rate of three parameters (i.e., migration rate, allele frequency and inbreeding coefficients) were between 20 to 60%. In addition, convergence of each MCMC run was diagnosed using Tracer v1.7.2 and Bayesian deviance calculated by R 4.1.0 software using R script authored by Meirmans [33] for determinant the best run. Finally, we used the results that set the value of deltaA = 0.20 and deltaF = 0.05, and MCMC runs performed 100,000,000 iterations with a burn-in of 10,000,000 iterations and a sampling frequency of 2000. PGDspider ver. 2.1.1.5 [34] was used to convert the data files for each software.

## 3. Results

### 3.1. Genetic Population Structure of Wild Boars in Fukushima Prefecture

**Figure 2:**
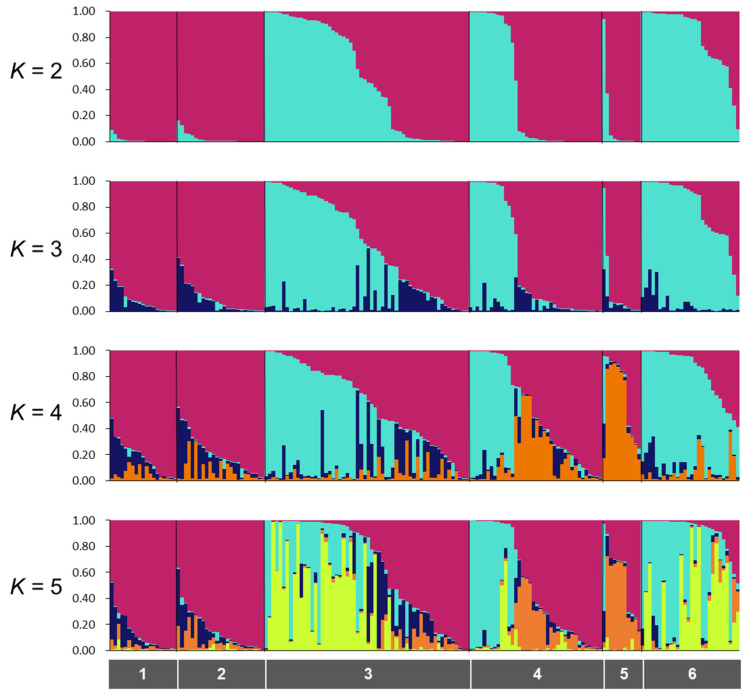
Results of the STRUCTURE analysis for *K* = 2–5. The gray color bars indicate region numbers (1, North of Soso; 2, South of Soso and Iwaki; 3, Ken-Poku; 4, Ken-Chu; 5, Ken-Nan; 6, Aizu). Location of each region has provided in Figure 1B.

We obtained 688 SNPs using MIG-seq analysis, in wild boar samples from Fukushima and Kumamoto Prefectures. The highest Δ*K* value was observed at *K* = 2 (Δ*K* = 687.88), followed by *K* = 3 (Δ*K* = 117.61) (Figure S2A). Therefore, the genetic lineages of the wild boar population in Fukushima and Kumamoto Prefectures could be classified into two or three groups (Figures S2A and S3). In addition, we obtained 328 SNPs from only wild boars in Fukushima Prefecture using MIG-seq analysis. STRUCTURE analysis, using 328 SNPs, showed that the highest Δ*K* value (Δ*K* = 1236.90) was obtained when *K* = 2 in comparison to other *K* values, strongly indicating that the wild boar population in Fukushima Prefecture could be classified into two groups (Figure 2 and Figure S2B). The bar plot for *K* = 2 showed two distinct clusters dominant in regions 1, 2 and 5 (the dark red cluster), and regions 3, 4 and 6 (dark red and geyser blue clusters) (Figure 2). To analyze the classification of wild boars in Fukushima Prefecture in detail, we carried out cluster analysis based on genetic similarities of wild boars inhabiting the six regions of Fukushima Prefecture. The analysis showed that wild boars in Fukushima Prefecture can be genetically classified into two groups as follows: The ones living in eastern and southern parts of Fukushima Prefecture (regions 1, 2 and 5); the ones inhabiting in north-central and western parts of Fukushima Prefecture (regions 3, 4 and 6) (Figure S4). Furthermore, we calculated *Fst* values among all six regions, and the result showed that range of significant *Fst* values were from 0.022 to 0.166 (*p* < 0.05, Table S2).

### 3.2. Geographical Fragmentation of Wild Boars in Fukushima Prefecture

**Figure 3:**
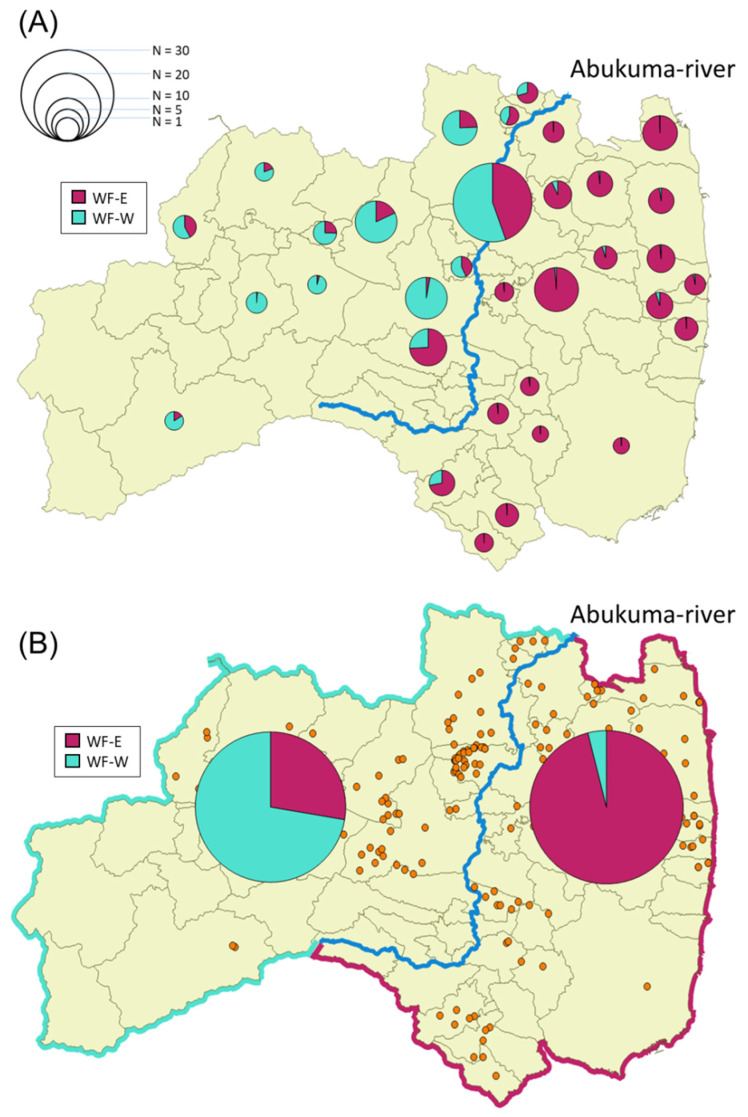
Sample number and detection rate (in percentage) of clusters WF-E and WF-W in each city/town/village (**A**) in eastern and western Abukuma River (**B**). The detection rate of clusters WF-E and WF-W was based on the results of STRUCTURE analysis (*K* = 2, Figure 2). The size of the circle in the graphs corresponds to the number of samples. Sampling points of wild boars are shown in orange dots.

As described above, wild boars inhabiting Fukushima Prefecture could be classified into two groups depending on the genetic analysis. We designated the dark red cluster as wild boars in Fukushima-East (WF-E) and the geyser blue cluster as wild boars in Fukushima-West (WF-W) based on the *K* = 2 data in Figure 2. For a detailed understanding of the distribution of these clusters in Fukushima Prefecture, the ratio of WF-E to WF-W was calculated in each municipality in Fukushima Prefecture (Figure 3A). A mixture of WF-E and WF-W was observed in pie charts for the municipalities along the Abukuma River (Figure 3A). Focusing on the genetic distribution of wild boars in terms of the separation by the Abukuma River, WF-E and WF-W tended to be dominant on the east side and the west side of the Abukuma River, respectively (Figure 3B). Moreover, analysis of molecular variance (AMOVA) results showed significant genetic differences between east and west groups of wild boars on the border of the Abukuma River (*p* ≤ 0.001), and the variation between the east and west groups represented 11% of the total variation (Table 1). Taken together, these indicated that the distribution of the two clusters tended to be divided between the eastern and western Abukuma River. From BayesAss analysis, values of the migration rates within the same side are almost same in both sides (0.967 in the east and 0.897 in the west). However, the migration rate in case from the east side population to west side population (0.103) is higher than in the reverse case (0.033).

## 4. Discussion

Our study revealed that there are two genetically differentiated lineages of wild boars (i.e., groups WF-E and WF-W) in Fukushima Prefecture. Inhabitation of wild boars in the west side in Fukushima prefecture (WF-W group) has been confirmed since 2004 [5]. With a simplistic point of view, the WF-W group of wild boars is considered to have originated from WF-E group. However, the significant genetic differentiation between groups of WF-E and WF-W, which is found in this study, is unlikely to have occurred in the less than 20 years. We will discuss the establishment of WF-W group of wild boars in Fukushima Prefecture as following sections.

When wild boars were divided into six regions based on their captured sites, the *Fst* values among each site were very small, indicating that the degree of genetic differentiation among the regions tended to be small (Table S2). Low levels of genetic differentiation observed here may be attributable to the characteristics of wild boars with comparatively low mobility and small home range [4,35]. Genetic lineages of wild boars seemed to be divided into two by the Abukuma River, suggesting that the Abukuma River restricts the migration of wild boars in Fukushima Prefecture (Figure 3). The Abukuma River is the largest river in Fukushima Prefecture, with a channel length of 239 km, flowing from south to north toward the neighboring Miyagi Prefecture (Figure S1A). Previous studies based on genetic analysis reported that rivers and valleys are responsible for the fragmentation of wild boar populations in Bulgaria [19] and Portugal [13,17]. However, rivers are not perfect barriers to the gene flow of wild boars because some individuals belonging to the same subpopulation were previously observed on both sides of a river [13,17]. Indeed, our study showed that the wild boars mainly distributed on the east side (cluster WF-E) were also found on the west side of the Abukuma River, and vice versa. Therefore, the Abukuma River itself may not be a complete barrier to the gene flow of wild boars in Fukushima Prefecture (Figure 3).

In general, the distribution of wild boar populations is considered to have been influenced by climate change, but also landscape change due to human activity, such as agriculture and forestry [3]. A study of genetic population structure in wild boars inhabiting the Gunma Prefecture [21] showed that landscape factors, such as main rivers, roads, urban areas, and train networks, can be boundaries for wild boar populations. Indeed, it has been shown that human infrastructures, such as highways, hinder wild boar migration [36,37]. Table S3 shows the land use in Fukushima Prefecture both as a whole and within 4 km of the riverbank in the municipalities bordering the Abukuma River. The urbanized area concentrates along the Abukuma River (Table S3) and highway, railroads, and the Shinkansen bullet train, run almost parallel to the Abukuma River (Figure S1), resulting in the division of the prefecture into eastern and western sides. Therefore, anthropogenic factors of landscape in the bordering area along the river may affect the migration and dispersal of wild boars.

According to the habitat distribution maps of wild boars throughout Japan, wild boars are generally not observed in areas with over 30 cm of snow accumulation continuing for more than 70 days in winter and those with 40% or less forest regions [5,38]. In Fukushima Prefecture, snow accumulation was relatively high in the west, which corresponds to the Aizu region (region 6 in Figure 1B), in comparison to other regions. According to wild boar distribution surveys conducted in 1981, 1993, and 2002, wild boars were not found in the west sides of the Abukuma River, including the Aizu region [5,38]. Since 2004, the inhabitation of wild boars has been confirmed even on the west side of the Abukuma River [5]. The increase of wild boar numbers on the west side of the prefecture was more concerned after the FDNPP accident. A recent study showed that wild boar is the most abundant species in the evacuation zone and was over three times more abundant in the evacuation order zone than in human-inhabited areas [39]. Therefore, was expected that the recent increase in the number of wild boars in the western Fukushima Prefecture occurred as a consequence of the increase in the number of wild boars inside the evacuation order zone after the FDNPP accident. Results by BayesAss analysis, in which the immigration rate of wild boars from east side population to west side population was high in comparison to the reverse case, provide some support for the above expectations. 

Our study showed that wild boars in the western Fukushima Prefecture were genetically different from those on the eastern side. This suggests that the establishment of the western wild boar population in Fukushima Prefecture may have occurred due to the migration of wild boars from neighboring prefectures rather than the eastern Fukushima Prefecture wild boar population. Indeed, nearly 70% of Fukushima Prefecture is made up of forest areas (Table S3), and the Abukuma Mountains, as well as the Ou Mountains, are located at the east and west side of the Abukuma River, respectively, straddling neighboring prefectures (Figure S1C). Such geomorphic characteristics may contribute to the easy migration of wild boars between Fukushima and neighboring prefectures. Therefore, it is necessary to clarify the genetic population structure of the wild boars not only within Fukushima Prefecture but also in the neighboring prefectures.

In general, population management for wild boars in Japan is mainly conducted under the control of units of the prefecture or each municipal; however, wild boar populations and their migration do not correspond to a particular administrative unit. As shown in this study, the genetic difference among wild boar populations reflects the population boundaries and the migration scale among populations; therefore, it is considered that the wild boar management based on the genetic population structure could be more effective. Furthermore, the long-term monitoring of the wild boar population is also important because the population dynamics of wild boars is likely to be affected by climate change, changes in human activities, and the landscape environment accompanied by depopulation in the future. In addition to these, the effect of long-term evacuation on the DRZ to the wild boar population is also an important issue. The long-term monitoring of wild boar populations after the Chernobyl nuclear power plant accident showed that the abundance of wild boars increased in the Chernobyl exclusion zone [40]. Therefore, it is necessary to continue long-term monitoring of wild boar population dynamics in Fukushima Prefecture, and monitoring methods based on genetic analysis, such as MIG-seq shown in this study, will provide information with sufficient resolution to understand the long-term dynamics and migration of the wild boar population. In addition, one aim of this study is to clarify that the wild boars containing high concentrations of radionuclides may be dispersed to western area of Fukushima Prefecture. Indeed, results from BayesAss analysis show that the immigration rate of wild boars from the east side population to the west side population was high in comparison to the reverse. However, it is difficult to discriminate whether the immigration of wild boar with high radioactive Cs into west of Fukushima Prefecture occurred one generation or over several generations. Therefore, it is important to study the relationships between genetic linages and radioactive Cs concentration in wild boars in the west side of Fukushima Prefecture. This will make clear the dynamics of radioactive Cs by migration of wild boar into the west area of Fukushima Prefecture.

## 5. Conclusions

We obtained 328 SNPs from wild boars in Fukushima Prefecture using MIG-seq analysis. Our study revealed that there are two genetically differentiated lineages of wild boars (i.e., groups WF-E and WF-W) and that both the Abukuma River and anthropogenic urbanization along the river may affect the migration and dispersal of wild boars. In addition, our results also suggest that the establishment of the western wild boar population in Fukushima Prefecture may have occurred due to the migration of wild boars from neighboring prefectures, in addition to the eastern population of Fukushima Prefecture.

## Figures and Tables

**Table 1:** Analysis of molecular variance (AMOVA) of two populations of wild boars divided to east and west side between Abukuma River in Fukushima Prefecture.

Source of Variation	Degrees of Freedom	Sum of Squares	Percentage of Variation	Fixation Index	
Among populations	1	764	11%	**0.115**	***
Among individuals within populations	177	5420	0%	−0.028	
Within individuals	179	5793	89%	**0.091**	***
Total	357	11,978	100%		

In bold significant values and significance levels are represented by: *** for *p* ≤ 0.001.

## Data Availability

Accession codes: Raw MIG-seq data are deposited at the DDBJ Sequence Read Archive (DRA) with accession number; DRA012666 (Submission), PRJDB12172 (BioProject), SAMD00399887–SAMD00400078 (BioSample), DRX305691–DRX305882 (Experiment) and DRR316310–DRR316501 (Run).

## Supplementary Materials

The following supporting information can be downloaded at: https://www.mdpi.com/article/10.3390/ani12040491/s1, Figure S1: Supplementary Figure S1. Geographical features (A), land use (B) and the location of the mountains (C) around Fukushima Prefecture. These figures were created using ArcGIS Pro (Esri Japan, Tokyo). The map of Japan, river, highway, railroad, land use and altitude were obtained by Ministry of Land, Infrastructure, Transport and Tourism (MLIT) of Japan (http://nlftp.mlit.go.jp/ksj/, accessed on 15 February 2022) area of difficult to return area, as of March 10th, 2020, was referred to the map released by Fukushima Prefecture (https://www.pref.fukushima.lg.jp/site/portal/list271-840.html, accessed on 15 February 2022).; Figure S2: Results of delta (Δ) *K* calculation based on Structure Harvester. (A) Result of Δ*K* from seven regions (six regions in Fukushima Prefecture and Kumamoto Prefecture). (B) Result of Δ*K* from six regions (six regions only in Fukushima Prefecture). Location of each region has provided in Figure 1A,B.; Figure S3: Results of the STRUCTURE analysis for *K* = 2–5. Gray bars at the bottom indicate region numbers (1, North of Soso; 2, South of Soso and Iwaki; 3, Ken-Poku; 4, Ken-Chu; 5, Ken-Nan; 6, Aizu in Fukushima Prefecture and 7, Kumamoto Prefecture). Location of each region has provided in Figure 1A,B.; Figure S4: Results of the cluster analysis for six regions of wild boars in Fukushima Prefecture (1, North of Soso; 2, South of Soso and Iwaki; 3, Ken-Poku; 4, Ken-Chu; 5, Ken-Nan; 6, Aizu in Fukushima Prefecture). Location of each region has provided in Figure 1B.; Table S1: Sample information.; Table S2: Pairwise *Fst* value (below) among the populations in Fukushima Prefecture based on 328 SNPs data.; Table S3: Land use of area of the Abukuma River basin and overall Fukushima Prefecture.
