# Supplementary material for: Genetic Population Structure of Wild Boars (Sus scrofa) in Fukushima Prefecture
Source: Animals (Basel). 2022 Feb 16;12(4):491. doi: 10.3390/ani12040491 (PMC8868446; doi:10.3390/ani12040491)
Supplement: Supplementary file 1 [file animals-12-00491-s001.zip › Supplyment File/Supplementary_files_FigureS1_S4_Saito_et_al.pdf]

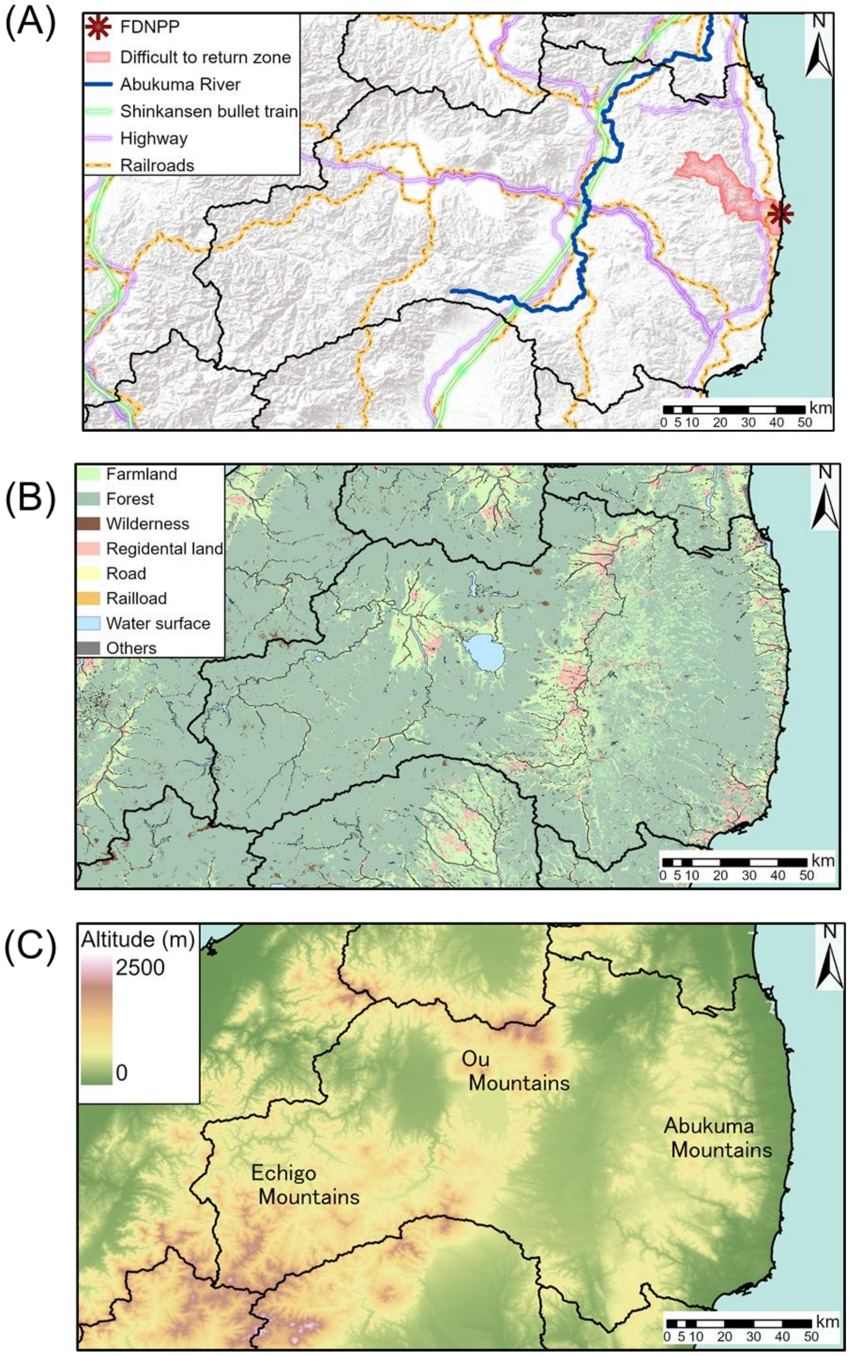

Supplementary Figure S1. Figure S1: Supplementary Figure S1. Geographical features (A), land use (B) and the location of the mountains (C) around Fukushima Prefecture. These figures were created using ArcGIS Pro (Esri Japan, Tokyo). The map of Japan, river, highway, railroad, land use and altitude were obtained by Ministry of Land, Infrastructure, Transport and Tourism (MLIT) of Japan (http://nlftp.mlit.go.jp/ksj/, accessed on 15 February 2022) area of difficult to return area, as of March 10th, 2020, was referred to the map released by Fukushima Prefecture (https://www.pref.fukushima.lg.jp/site/portal/list271-840.html, accessed on 15 February 2022) .

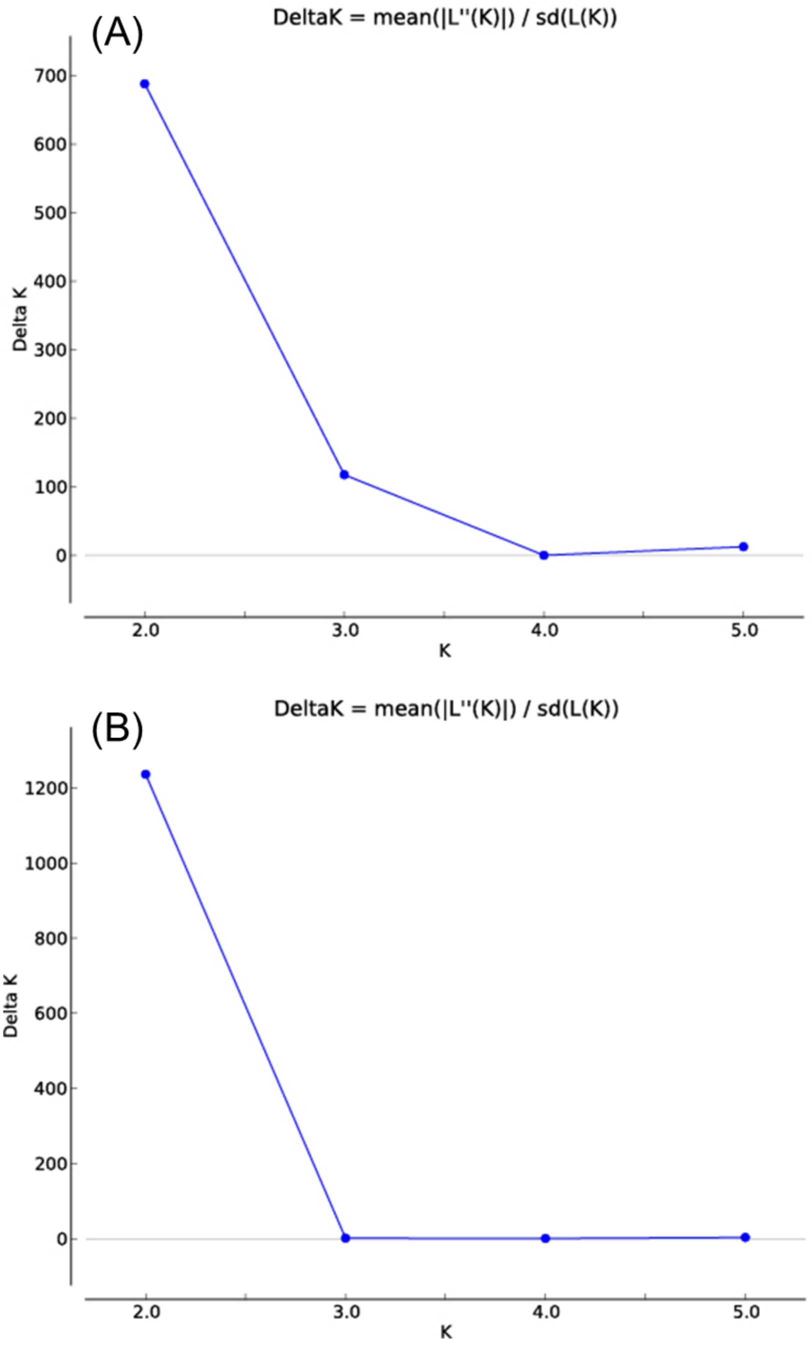

Supplementary Figure S2. Results of delta (Δ) *K* calculation based on Structure Harvester. (A) Result of Δ*K* from seven regions (six regions in Fukushima Prefecture and Kumamoto Prefecture). (B) Result of Δ*K* from six regions (six regions only in Fukushima Prefecture). Location of each region has provided in Figure 1A and 1B.

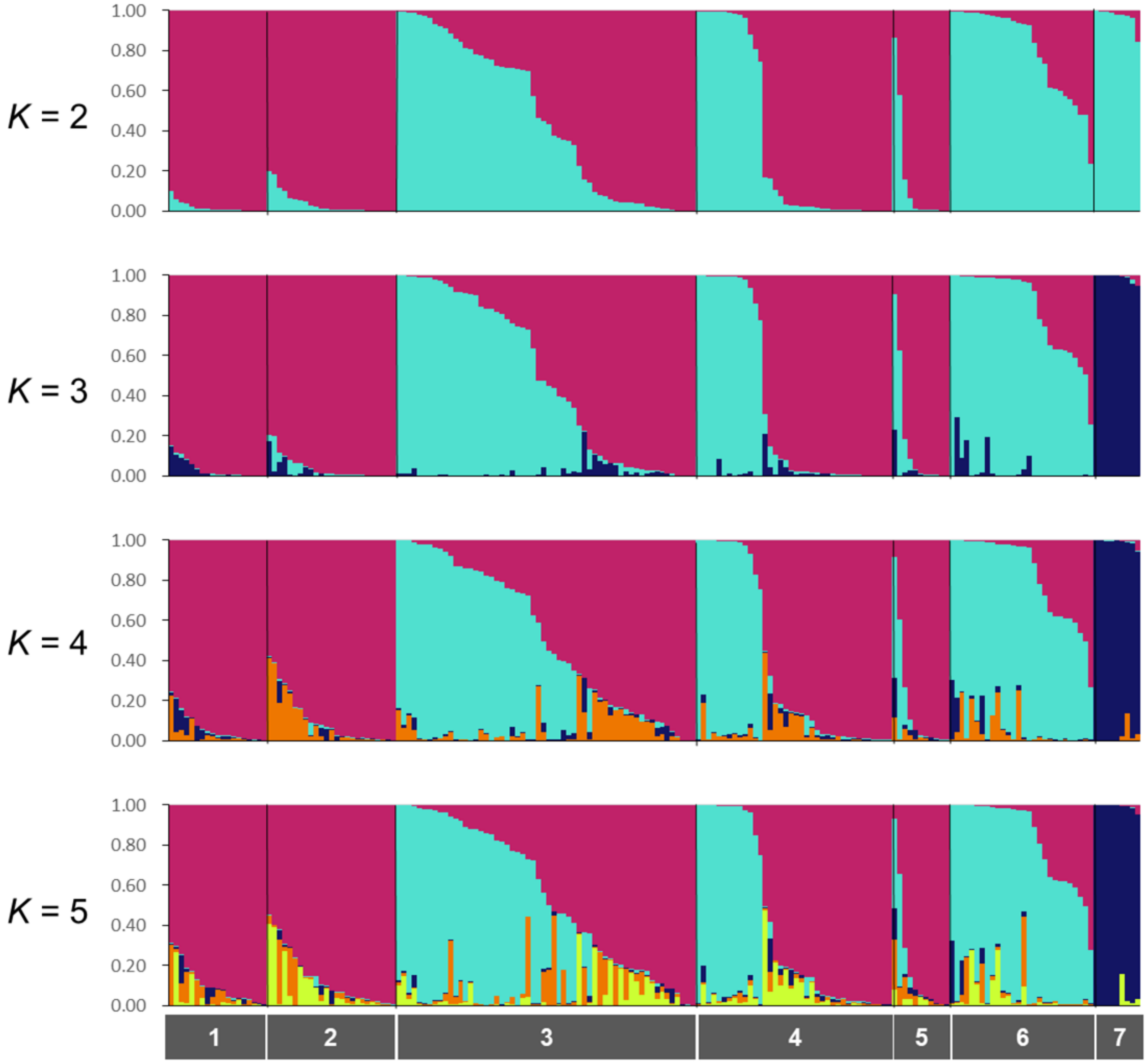

Supplementary Figure S3: Results of the STRUCTURE analysis for *K* = 2-5. Gray bars at the bottom indicate region numbers (1, North of Soso; 2, South of Soso and Iwaki; 3, Ken-Poku; 4, Ken-Chu; 5, Ken-Nan; 6, Aizu in Fukushima Prefecture and 7, Kumamoto Prefecture). Location of each region has provided in Figure 1A and 1B.

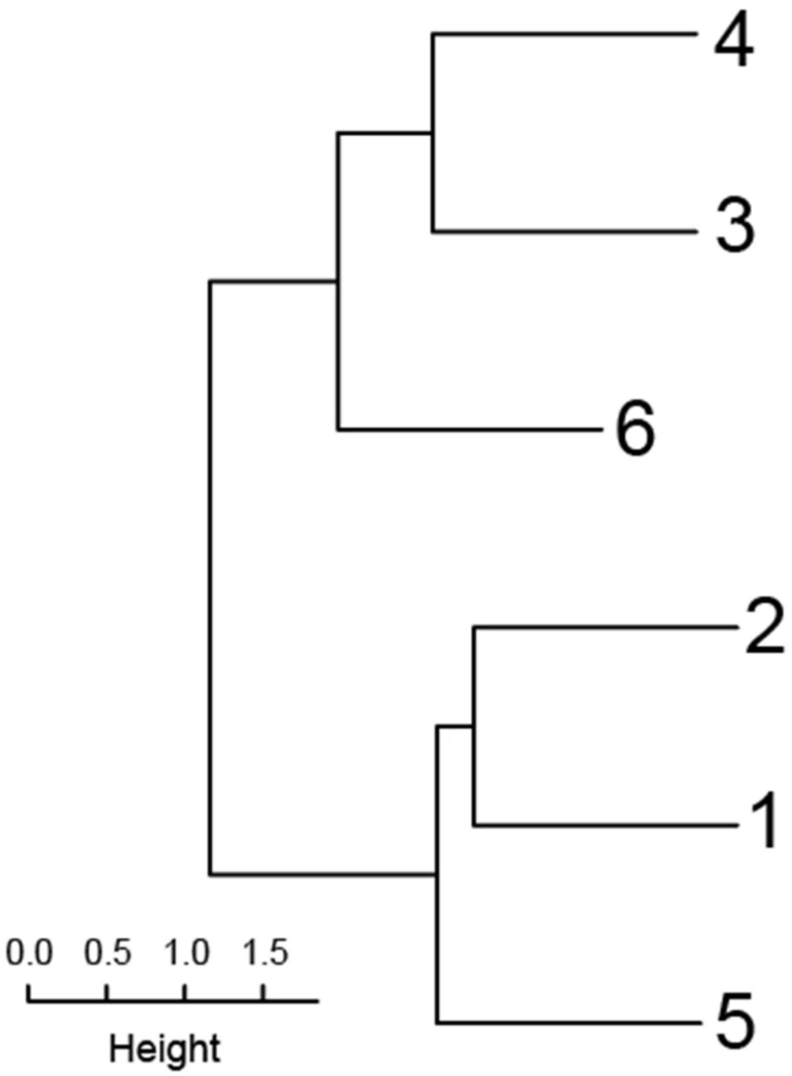

Supplementary Figure S4: Results of the cluster analysis for six regions of wild boars in Fukushima Prefecture (1, North of Soso; 2, South of Soso and Iwaki; 3, Ken-Poku; 4, Ken-Chu; 5, Ken-Nan; 6, Aizu in Fukushima Prefecture). Location of each region has provided in Figure 1B.
