# Supplementary material for: Genetic Population Structure of Wild Boars (Sus scrofa) in Fukushima Prefecture
Source: Animals (Basel). 2022 Feb 16;12(4):491. doi: 10.3390/ani12040491 (PMC8868446; doi:10.3390/ani12040491)
Supplement: Supplementary file 1 [file animals-12-00491-s001.zip › Supplyment File/Supplementary_files_TableS2_S3_Saito_et_al.pdf]

Supplementary Table S2 Pairwise *Fst* value (below) among the populations in Fukushima Prefecture based on 328 SNPs data.

Number of individual	19	25	58	38	11	28
Population Number	1	2	3	4	5	6
1						
2	0.006					
3	**0.057****	**0.042****				
4	**0.034***	**0.022***	0.008			
5	**0.073***	**0.069****	**0.087****	**0.050***		
6	**0.163****	**0.142****	**0.041****	**0.057****	**0.166****	

Pairwise *F*st value calculated using an AMOVA approach.

In bold significant values and significance levels are represented by: * for $P \leq 0.05$, **$P \leq 0.01$

Supplementary Table S3 Land use of area of Abukuma river basin[*1] and overall Fukushima Prefecture.

Land use	Area of Abukuma river Basin[*1]				Overall Fukushima Prefecture[*2]	
	Left Bank		Right Bank			
	Area (km^2)	Rate	Area (km^2)	Rate	Area (km^2)	Rate
Farmland	185	0.39	194	0.40	1,407	0.10
Forest	140	0.30	201	0.42	9,728	0.71
Wilderness	3	0.01	4	0.01	69	0.01
Water surface (River, Waterway etc.)	13	0.03	11	0.02	457	0.03
Road	2	0.01>	1>	0.01>	533	0.04
Residential land	111	0.24	60	0.12	505	0.04
Others	17	0.04	14	0.03	1,084	0.08
Total	471	1.00	485	1.00	13,783	1.00

[*1] Land use within 4 km of the riverbank in municipalities in Fukushima Prefecture bordering the Abukuma River basin

[*2] Refer to the current status of land use in Fukushima Prefecture in 2018.
(https://www.pref.fukushima.lg.jp/sec/11015b/fukushimaken-tochi-riyou-genkyou.html, accessed on 15 February 2022)
