# Supplementary material for: Genetic Population Structure of Wild Boars (Sus scrofa) in Fukushima Prefecture
Source: Animals (Basel). 2022 Feb 16;12(4):491. doi: 10.3390/ani12040491 (PMC8868446; doi:10.3390/ani12040491)
Supplement: Supplementary file 1 [file animals-12-00491-s001.zip › Supplyment File/Supplementary_files_Table_S1_Saito_et_al.pdf]

Supplementary Table S1 Sample information.

Sample ID	Local distriction[*1]	East/West side of Abukuma river*[2]	Prefecture	City/Town/Villege	Captured date	Gender	DRX Number of DDBJ
WF001	1	East	Fukushima	Minamisoma	2016/6/11	Female	DRX305691
WF002	3	West	Fukushima	Fukushima	2016/6/16	Male	DRX305692
WF003	1	East	Fukushima	Soma	2016/6/17	Female	DRX305693
WF004	3	West	Fukushima	Fukushima	2016/6/21	Male	DRX305694
WF005	1	East	Fukushima	Soma	2016/6/20	Female	DRX305695
WF006	3	East	Fukushima	Kawamata	2016/6/20	Male	DRX305696
WF009	3	East	Fukushima	Kawamata	2016/6/29	Male	DRX305699
WF010	3	East	Fukushima	Kawamata	2016/7/1	Female	DRX305700
WF011	6	West	Fukushima	Minamiaizu	2016/7/10	Male	DRX305701
WF012	3	West	Fukushima	Motomiya	2013/10/17	Male	DRX305702
WF013	4	East	Fukushima	Tamura	2013/11/1	Male	DRX305703
WF014	4	East	Fukushima	Sukagawa	2016/7/17	Male	DRX305704
WF015	1	East	Fukushima	Soma	2016/7/15	Female	DRX305705
WF016	6	West	Fukushima	Inawashiro	2016/7/21	Male	DRX305706
WF017	6	West	Fukushima	Inawashiro	2016/7/26	Male	DRX305707
WF018	1	East	Fukushima	Soma	2016/7/26	Male	DRX305708
WF019	1	East	Fukushima	Minamisoma	2016/7/31	Male	DRX305709
WF020	6	West	Fukushima	Inawashiro	2016/8/4	Male	DRX305710
WF021	3	East	Fukushima	Date	2016/8/17	Male	DRX305711
WF022	4	West	Fukushima	Koriyama	2016/8/23	Male	DRX305712
WF023	4	West	Fukushima	Koriyama	2016/8/24	Female	DRX305713
WF024	6	West	Fukushima	Inawashiro	2016/8/29	Female	DRX305714
WF025	4	East	Fukushima	Ishikawa	2016/9/4	Male	DRX305715
WF026	3	West	Fukushima	Fukushima	2016/9/9	Male	DRX305716
WF027	1	East	Fukushima	Minamisoma	2016/9/13	Male	DRX305717
WF028	6	West	Fukushima	Aizumisato	2016/9/13	Female	DRX305718
WF029	6	West	Fukushima	Minamiaizu	2016/9/16	Male	DRX305719
WF030	4	East	Fukushima	Tamura	2013/11/11	Female	DRX305720
WF031	1	East	Fukushima	Iitate	2013/11/8	Male	DRX305721
WF032	1	East	Fukushima	Iitate	2013/11/7	Male	DRX305722
WF033	4	East	Fukushima	Tamura	2013/11/16	Unknown	DRX305723
WF034	1	East	Fukushima	Minamisoma	2013/11/20	Male	DRX305724
WF035	1	East	Fukushima	Iitate	2013/11/27	Male	DRX305725
WF036	4	East	Fukushima	Tamura	2013/12/7	Female	DRX305726
WF037	6	West	Fukushima	Kitakata	2014/1/11	Female	DRX305727
WF038	3	West	Fukushima	Fukushima	2014/1/22	Male	DRX305728
WF039	4	East	Fukushima	Tamura	2014/1/24	Female	DRX305729
WF040	1	East	Fukushima	Iitate	2014/2/11	Male	DRX305730
WF041	3	East	Fukushima	Date	2014/3/3	Male	DRX305731
WF042	5	East	Fukushima	Hanawa	2016/9/19	Male	DRX305732
WF043	5	East	Fukushima	Hanawa	2016/9/18	Male	DRX305733
WF044	1	East	Fukushima	Minamisoma	2016/9/18	Male	DRX305734
WF045	1	East	Fukushima	Soma	2016/9/26	Male	DRX305735
WF046	5	East	Fukushima	Hanawa	2016/9/20	Male	DRX305736
WF047	3	West	Fukushima	Fukushima	2016/9/25	Male	DRX305737
WF048	6	West	Fukushima	Aizumisato	2016/10/4	Male	DRX305738
WF049	4	East	Fukushima	Tamura	2016/10/7	Male	DRX305739
WF050	3	West	Fukushima	Fukushima	2016/9/23	Male	DRX305740
WF051	4	West	Fukushima	Koriyama	2016/10/12	Male	DRX305741
WF052	6	West	Fukushima	Inawashiro	2016/10/23	Male	DRX305742
WF053	4	East	Fukushima	Tamura	2016/10/21	Male	DRX305743
WF054	4	East	Fukushima	Tamura	2016/10/22	Male	DRX305744
WF055	4	East	Fukushima	Sukagawa	2016/10/30	Female	DRX305745
WF056	3	West	Fukushima	Nihonmatsu	2016/10/30	Male	DRX305746
WF057	3	West	Fukushima	Nihonmatsu	2016/10/30	Female	DRX305747
WF058	3	West	Fukushima	Nihonmatsu	2016/10/30	Female	DRX305748
WF059	3	West	Fukushima	Kunimi	2016/10/30	Male	DRX305749
WF060	1	East	Fukushima	Soma	2016/10/29	Female	DRX305750
WF061	1	East	Fukushima	Soma	2013/10/31	Male	DRX305751
WF062	6	West	Fukushima	Inawashiro	2016/11/3	Male	DRX305752
WF063	4	East	Fukushima	Tamura	2016/11/2	Male	DRX305753
WF064	3	West	Fukushima	Nihonmatsu	2016/11/4	Female	DRX305754
WF065	3	West	Fukushima	Nihonmatsu	2016/11/7	Female	DRX305755

Sample ID	Local distriction[*1]	East/West side of Abukuma river*[2]	Prefecture	City/Town/Villege	Captured date	Gender	DRX Number of DDBJ
WF066	6	West	Fukushima	Inawashiro	2016/11/9	Male	DRX305756
WF067	4	East	Fukushima	Tamura	2016/11/9	Male	DRX305757
WF068	6	West	Fukushima	Bandai	2016/11/10	Female	DRX305758
WF069	4	East	Fukushima	Miharu	2016/11/9	Female	DRX305759
WF070	4	East	Fukushima	Miharu	2016/11/10	Male	DRX305760
WF071	4	East	Fukushima	Sukagawa	2016/11/14	Male	DRX305761
WF072	4	East	Fukushima	Sukagawa	2016/11/15	Female	DRX305762
WF073	4	West	Fukushima	Sukagawa	2016/11/18	Female	DRX305763
WF074	2	East	Fukushima	Iwaki	2016/11/23	Female	DRX305764
WF075	5	East	Fukushima	Hanawa	2016/11/23	Male	DRX305765
WF076	3	West	Fukushima	Nihonmatsu	2016/11/28	Female	DRX305766
WF077	4	West	Fukushima	Sukagawa	2016/12/16	Female	DRX305767
WF078	1	East	Fukushima	Iitate	2017/6/7	Female	DRX305768
WF079	6	West	Fukushima	Inawashiro	2017/6/20	Male	DRX305769
WF080	6	West	Fukushima	Inawashiro	2017/6/26	Male	DRX305770
WF081	1	East	Fukushima	Soma	2017/6/25	Male	DRX305771
WF082	2	East	Fukushima	Hirata	2017/6/28	Female	DRX305772
WF083	4	West	Fukushima	Koriyama	2017/6/12	Male	DRX305773
WF084	4	East	Fukushima	Sukagawa	2017/7/3	Male	DRX305774
WF085	3	West	Fukushima	Fukushima	2017/7/17	Female	DRX305775
WF086	4	West	Fukushima	Koriyama	2017/7/18	Female	DRX305776
WF087	6	West	Fukushima	Aizuwakamatsu	2017/7/26	Male	DRX305777
WF088	4	West	Fukushima	Koriyama	2017/7/29	Male	DRX305778
WF089	4	West	Fukushima	Koriyama	2017/8/6	Female	DRX305779
WF090	6	West	Fukushima	Aizumisato	2017/9/7	Female	DRX305780
WF091	1	East	Fukushima	Soma	2017/9/6	Male	DRX305781
WF092	6	West	Fukushima	Aizuwakamatsu	2017/9/13	Female	DRX305782
WF093	4	West	Fukushima	Koriyama	2017/9/21	Male	DRX305783
WF094	5	East	Fukushima	Yamatsuri	2017/9/27	Male	DRX305784
WF095	3	East	Fukushima	Date	2017/10/4	Male	DRX305785
WF096	3	West	Fukushima	Motomiya	2017/10/6	Male	DRX305786
WF097	6	West	Fukushima	Inawashiro	2017/10/23	Female	DRX305787
WF098	4	East	Fukushima	Ishikawa	2017/11/2	Male	DRX305788
WF099	6	West	Fukushima	Bandai	2017/11/5	Male	DRX305789
WF100	4	East	Fukushima	Tamura	2017/11/6	Female	DRX305790
WF101	2	East	Fukushima	Hirata	2017/11/20	Male	DRX305791
WF102	5	East	Fukushima	Yamatsuri	2017/11/26	Male	DRX305792
WF103	6	West	Fukushima	Nishiaizu	2015/1/16	Male	DRX305793
WF104	6	West	Fukushima	Nishiaizu	2015/1/29	Male	DRX305794
WF105	4	East	Fukushima	Tamura	2015/2/6	Female	DRX305795
WF106	6	West	Fukushima	Nishiaizu	2015/2/5	Female	DRX305796
WF107	4	East	Fukushima	Tamura	2016/11/9	Male	DRX305797
WF108	3	East	Fukushima	Motomiya	2015/3/7	Male	DRX305798
WF109	2	East	Fukushima	Katsurao	2015/2/27	Male	DRX305799
WF110	2	East	Fukushima	Katsurao	2015/3/6	Male	DRX305800
WF111	3	West	Fukushima	Koori	2015/5/10	Female	DRX305801
WF112	3	West	Fukushima	Koori	2015/6/15	Female	DRX305802
WF113	6	West	Fukushima	Bandai	2015/6/17	Male	DRX305803
WF114	4	East	Fukushima	Ishikawa	2015/6/27	Male	DRX305804
WF115	3	West	Fukushima	Fukushima	2015/7/3	Male	DRX305805
WF116	5	East	Fukushima	Tanagura	2015/6/29	Female	DRX305806
WF117	5	East	Fukushima	Tanagura	2015/6/30	Male	DRX305807
WF118	5	East	Fukushima	Tanagura	2015/7/6	Male	DRX305808
WF119	5	East	Fukushima	Tanagura	2015/7/17	Male	DRX305809
WF120	6	West	Fukushima	Inawashiro	2015/8/9	Female	DRX305810
WF121	5	East	Fukushima	Tanagura	2015/9/6	Male	DRX305811
WF122	6	West	Fukushima	Bandai	2015/10/4	Male	DRX305812
WF123	6	West	Fukushima	Kitakata	2016/1/24	Female	DRX305813
WF124	6	West	Fukushima	Nishiaizu	2016/2/8	Male	DRX305814
WF125	3	West	Fukushima	Kunimi	2016/2/12	Male	DRX305815
WF126	3	West	Fukushima	Kunimi	2016/5/22	Female	DRX305816
WF127	4	West	Fukushima	Koriyama	2016/5/27	Male	DRX305817
WF128	4	West	Fukushima	Koriyama	2016/6/11	Female	DRX305818
WF129	3	West	Fukushima	Fukushima	2016/6/10	Female	DRX305819

Sample ID	Local distriction[*1]	East/West side of Abukuma river*[2]	Prefecture	City/Town/Villege	Captured date	Gender	DRX Number of DDBJ
WF130	3	East	Fukushima	Kawamata	2016/6/14	Male	DRX305820
WF131	4	West	Fukushima	Koriyama	2016/6/22	Unknown	DRX305821
WF132	2	East	Fukushima	Futaba	2016/2/15	Female	DRX305822
WF133	2	East	Fukushima	Okuma	2016/2/15	Female	DRX305823
WF134	2	East	Fukushima	Futaba	2016/2/17	Female	DRX305824
WF135	2	East	Fukushima	Okuma	2016/2/17	Male	DRX305825
WF136	2	East	Fukushima	Okuma	2016/2/19	Female	DRX305826
WF137	2	East	Fukushima	Okuma	2016/2/19	Male	DRX305827
WF138	2	East	Fukushima	Tomioka	2016/2/19	Female	DRX305828
WF139	2	East	Fukushima	Tomioka	2016/2/19	Female	DRX305829
WF140	2	East	Fukushima	Namie	2016/2/24	Male	DRX305830
WF141	2	East	Fukushima	Namie	2016/2/24	Female	DRX305831
WF142	2	East	Fukushima	Namie	2016/2/24	Female	DRX305832
WF143	2	East	Fukushima	Katsurao	2016/2/24	Male	DRX305833
WF144	2	East	Fukushima	Tomioka	2016/2/23	Male	DRX305834
WF145	2	East	Fukushima	Tomioka	2016/2/23	Male	DRX305835
WF146	2	East	Fukushima	Namie	2016/2/22	Male	DRX305836
WF147	2	East	Fukushima	Namie	2016/2/22	Male	DRX305837
WF148	3	East	Fukushima	Kawamata	2016/6/16	Male	DRX305838
WF149	3	East	Fukushima	Kawamata	2016/6/17	Male	DRX305839
WF150	2	East	Fukushima	Furudono	2016/9/12	Male	DRX305840
WF151	4	East	Fukushima	Sukagawa	2016/9/17	Male	DRX305841
WF152	3	West	Fukushima	Nihonmatsu	2016/10/28	Female	DRX305842
WF153	3	West	Fukushima	Nihonmatsu	2016/11/4	Male	DRX305843
WF154	3	West	Fukushima	Nihonmatsu	2016/11/16	Female	DRX305844
WF155	4	East	Fukushima	Sukagawa	2016/11/24	Female	DRX305845
WF156	3	East	Fukushima	Nihonmatsu	2018/5/9	Female	DRX305846
WF158	3	East	Fukushima	Nihonmatsu	2018/5/14	Female	DRX305848
WF160	2	East	Fukushima	Namie	2018/1/30	Male	DRX305850
WF161	2	East	Fukushima	Futaba	2018/2/5	Female	DRX305851
WF162	2	East	Fukushima	Katsurao	2018/2/22	Female	DRX305852
WF163	3	West	Fukushima	Nihonmatsu	2017/12/30	Male	DRX305853
WF164	3	West	Fukushima	Nihonmatsu	2017/12/30	Male	DRX305854
WF165	3	West	Fukushima	Nihonmatsu	2018/1/4	Male	DRX305855
WF166	3	West	Fukushima	Nihonmatsu	2018/1/5	Male	DRX305856
WF167	3	West	Fukushima	Nihonmatsu	2018/1/8	Male	DRX305857
WF168	3	West	Fukushima	Nihonmatsu	2018/1/11	Male	DRX305858
WF169	3	West	Fukushima	Nihonmatsu	2018/1/11	Female	DRX305859
WF170	3	West	Fukushima	Nihonmatsu	2018/1/12	Male	DRX305860
WF171	3	West	Fukushima	Nihonmatsu	2018/1/13	Male	DRX305861
WF172	3	West	Fukushima	Nihonmatsu	2018/1/15	Female	DRX305862
WF173	3	West	Fukushima	Nihonmatsu	2018/1/18	Female	DRX305863
WF174	3	West	Fukushima	Nihonmatsu	2018/1/18	Male	DRX305864
WF175	3	West	Fukushima	Nihonmatsu	2018/1/18	Female	DRX305865
WF176	3	West	Fukushima	Nihonmatsu	2017/9/8	Female	DRX305866
WF177	3	West	Fukushima	Nihonmatsu	2018/1/22	Male	DRX305867
WF178	3	West	Fukushima	Nihonmatsu	2018/2/9	Male	DRX305868
WF179	3	West	Fukushima	Nihonmatsu	2018/2/11	Male	DRX305869
WF180	3	West	Fukushima	Nihonmatsu	2018/2/26	Male	DRX305870
WF181	3	West	Fukushima	Nihonmatsu	2018/3/7	Female	DRX305871
WF182	3	West	Fukushima	Nihonmatsu	2018/3/8	Female	DRX305872
WF183	3	West	Fukushima	Nihonmatsu	2018/3/17	Female	DRX305873
WF184	7	-	Kumamoto	-	2018/7/9	Male	DRX305874
WF185	7	-	Kumamoto	-	2018/7/11	Female	DRX305875
WF186	7	-	Kumamoto	-	2018/7/15	Male	DRX305876
WF187	7	-	Kumamoto	-	2018/7/15	Female	DRX305877
WF188	7	-	Kumamoto	-	2018/7/16	Female	DRX305878
WF189	7	-	Kumamoto	-	2018/7/19	Male	DRX305879
WF190	7	-	Kumamoto	-	2018/7/19	Female	DRX305880
WF191	7	-	Kumamoto	-	2018/7/21	Male	DRX305881
WF192	7	-	Kumamoto	-	2018/7/21	Male	DRX305882

[*1] Define by Figure 1B.
[*2] Define by captureed from east or west side of the Abukuma River.
